# High frequencies of theropod bite marks provide evidence for feeding, scavenging, and possible cannibalism in a stressed Late Jurassic ecosystem

**DOI:** 10.1371/journal.pone.0233115

**Published:** 2020-05-27

**Authors:** Stephanie K. Drumheller, Julia B. McHugh, Miriam Kane, Anja Riedel, Domenic C. D’Amore

**Affiliations:** 1 Department of Earth and Planetary Sciences, The University of Tennessee, Knoxville, TN, United States of America; 2 Museums of Western Colorado, Grand Junction, CO, United States of America; 3 Department of Physical and Environmental Sciences, Colorado Mesa University, Grand Junction, CO, United States of America; 4 Department of Natural Sciences, Daemen College, Amherst, NY, United States of America; University of Wisconsin Madison, UNITED STATES

## Abstract

Bite marks provide direct evidence for trophic interactions and competition in the fossil record. However, variations in paleoecological dynamics, such as trophic relationships, feeding behavior, and food availability, govern the frequency of these traces. Theropod bite marks are particularly rare, suggesting that members of this clade might not often focus on bone as a resource, instead preferentially targeting softer tissues. Here, we present an unusually large sample of theropod bite marks from the Upper Jurassic Mygatt-Moore Quarry (MMQ). We surveyed 2,368 vertebrate fossils from MMQ in this analysis, with 684 specimens (28.885% of the sample) preserving at least one theropod bite mark. This is substantially higher than in other dinosaur-dominated assemblages, including contemporaneous localities from the Morrison Formation. Observed bite marks include punctures, scores, furrows, pits, and striations. Striated marks are particularly useful, diagnostic traces generated by the denticles of ziphodont teeth, because the spacing of these features can be used to provide minimum estimates of trace maker size. In the MMQ assemblage, most of the striations are consistent with denticles of the two largest predators known from the site: *Allosaurus* and *Ceratosaurus*. One of the bite marks suggests that a substantially larger theropod was possibly present at the site and are consistent with large theropods known from other Morrison Formation assemblages (either an unusually large *Allosaurus* or a separate, large-bodied taxon such as *Saurophaganax* or *Torvosaurus*). The distribution of the bite marks on skeletal elements, particularly those found on other theropods, suggest that they potentially preserve evidence of scavenging, rather than active predation. Given the relative abundances of the MMQ carnivores, partnered with the size-estimates based on the striated bite marks, the feeding trace assemblage likely preserves the first evidence of cannibalism in *Allosaurus*.

## Introduction

Bite marks provide insight into several behaviors of extinct animals, including trophic interactions, feeding strategy, prey selection, and even intraspecific competition [e.g., 1–5]. However, those insights often are only possible when individual traces can be associated with a specific actor. Correlation of bite marks with actors is challenging, especially when diagnostic trace types represent a small proportion of the total number of bone surface modifications [e.g., 4, 6–8], when trace types between actors are convergent [9, 10], and when similar trace makers inhabit the same environment [e.g., 1, 11, 12].

Theropod bite marks are particularly rare in fossil assemblages [13], with tooth marked bones reported to represent ≤4.0% of non-avian dinosaur dominated assemblages, a significantly lower rate than the 13.1 to 37.5% expected frequencies of mammalian modified bones [*sensu* 14]. Among theropod bite marks, actualistic research predicts that roughly 5.0% of bite marks left by predators with ziphodont dentition will leave striations, linear features formed when the individual denticles of a serrated tooth leave distinct traces [7]. These traces are exceedingly rare in dinosaurian assemblages [15], but when they are available, they can be particularly useful in taxon identification and trace maker body size estimates [16].

Of known theropod tooth marks, descriptions of tyrannosaur bite marks are disproportionately overrepresented in the literature, perhaps owing as much to the species’ adaptations for osteophagy as to its general popularity [e.g., 17–20]. Traces from other taxa are less frequently described and more poorly known [e.g., 12, 21]. Among these other theropods, taxa from the Upper Jurassic Morrison Formation are the best studied [e.g., 12, 22–25]. However, a high diversity of theropods preserved within the Morrison depositional system, partnered with similarities in trace types made by these clades, has made association of these marks with specific actors difficult [12].

Here, we present an unusually high density of theropod bite marks from the Upper Jurassic Mygatt-Moore Quarry (MMQ) in Colorado, U.S.A. The large number of bite marks provides a rare opportunity to test methods for differentiating trace makers and characterize their body size using measurements of striated marks. Conclusions drawn from these rare traces provide insights into the trophic dynamics and feeding ecology of theropods in the MMQ.

### Institutional abbreviations

Museums of Western Colorado (MWC); Sam Noble Oklahoma Museum of Natural History (OMNH); Utah Museum of Natural History (UMNH).

## Materials and methods

### Geologic setting

**Fig 1:**
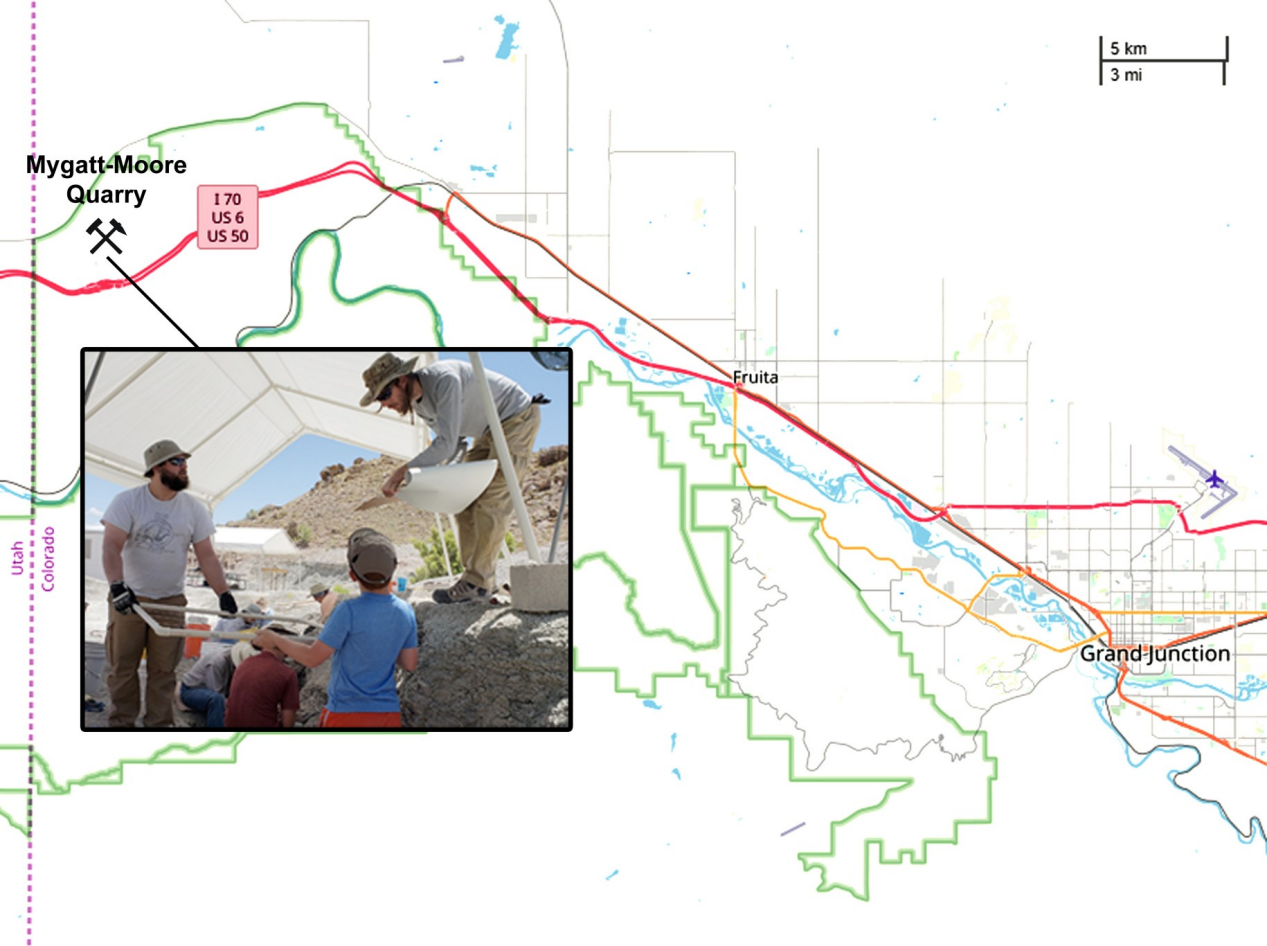
Map of western Colorado showing the location of the Mygatt-Moore Quarry (© OpenStreetMap contributors | https://www.openstreetmap.org/copyright). Inset photo shows museum field crew and citizen scientists during a public excavation through the Museums of Western Colorado at the Mygatt-Moore Quarry in 2018. The individuals pictured here provided written informed consent (as outlined in PLoS consent form) to publish their image alongside the manuscript.

The Mygatt-Moore Quarry (MMQ) is a dinosaur-dominated assemblage within the Upper Jurassic Morrison Formation (Brushy Basin Member) and is located within the McInnis Canyons National Conservation Area near the Utah-Colorado border (Fig 1). Discovered in 1981 by friends Pete and Marilyn Mygatt and J.D. and Vanetta Moore while hiking, the site is co-managed by the Museums of Western Colorado (MWC) and the Bureau of Land Management (BLM). Over thirty years of excavations by crews from the MWC and the Dinamation International Society have facilitated the recovery of thousands of vertebrate fossils, including the holotype specimens of *Hulettia hawesi* (Osteichthyes, Halecostomi) [26], *Morrolepis schaefferi* (Osteichthyes, Dipnoi) [26], and *Mymoorapelta maysi* (Ornithischia, Ankylosauria) [27].

The fossil-bearing horizon is a 1–2 meter-thick unit exposed within laminated to medium bedded grey, silty mudstones within the middle part of the Brushy Basin Member of the Morrison Formation [27–31]. Radiometric analysis of ash-fall zircons from the quarry has returned an age of 152.18 ± 0.29 Ma, bounding the Kimmeridgian and Tithonian stages of the Late Jurassic Period [32].

The MMQ is interpreted to preserve a riparian ecosystem with abundant vegetation and a high water table, but without continuous standing water [28–30, 32, 33]. The site preserves abundant carbonized plant material, but crocodylomorph, turtle, fish, and aquatic invertebrate remains are rare in the main horizon, indicating a lack of perennial standing water at the site [29, 30]. Previous taphonomic work has demonstrated that this as an autochthonous assemblage within an attritional deposit in an overbank setting with very few articulated specimens (ratio of articulated specimens at the site is 0.00337), no preferred orientation of skeletal elements, and a large proportion of fairly complete elements within the assemblage [29, 30].

### Bite mark and trace maker identification

**Fig 2:**
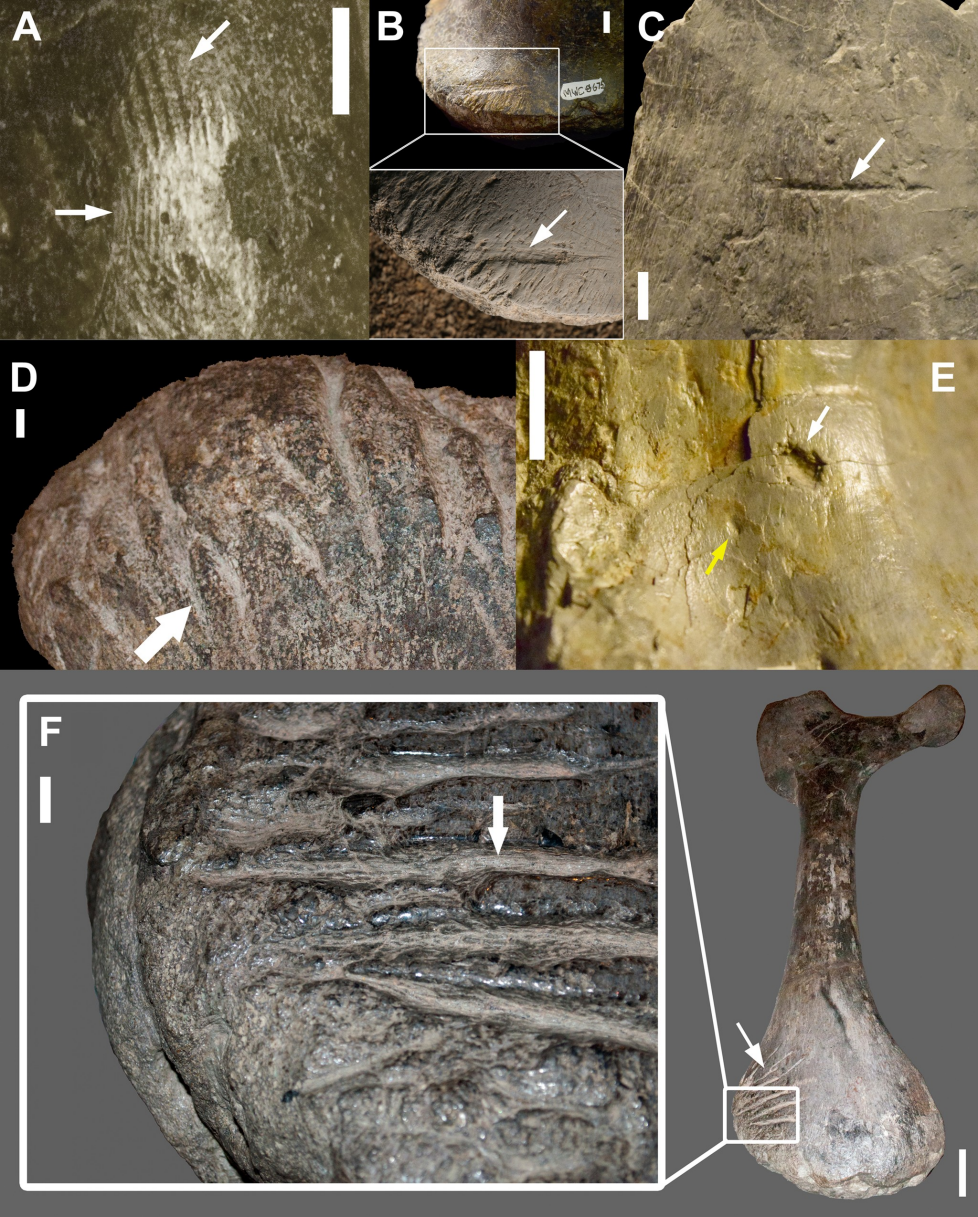
Types of bite marks observed in the MMQ assemblage with arrows indicating features of note. A, striated marks produced by ziphodont tooth on an *Allosaurus* sp. pedal claw (MWC 7263); B, a striated score on an *Allosaurus* sp. vertebral centrum (MWC 8675); C, a score on an *Apatosaurus* sp. rib fragment (MWC 3853); D, a dense cluster of furrows on a distal *Apatosaurus* sp. pubis (MWC 861); E, a puncture (white arrow) and a pit (yellow arrow) on an *Allosaurus* sp. caudal vertebral centrum; F, a dense cluster of striated furrows *Apatosaurus* sp. ischium (MWC 4011). All scale bars equal 10 mm.

Thousands of vertebrate fossils have been collected from the Mygatt-Moore Quarry (MMQ) over decades of work, and as excavations at the site are ongoing, the total number of fossils from the site housed by the Museums of Western Colorado (MWC) is constantly changing. We surveyed 2,368 fossil specimens, which as of the winter of early 2020 included all specimens not still under preparation in the MWC paleontology lab and specimens on exhibit or loan. Fossil teeth were also excluded from this study. We inspected specimens for bone surface modifications using raking light and low magnification, following the methods outlined in Blumenschine et al. [34]. Bite marks were measured (overall length vs. width) and identified based on the criteria of Binford [35] in that they must exhibit evidence of crushing or impact damage. These features were further classified, again following Binford [35], into pits (indentations that do not pierce the cortical bone), punctures (indentations that do pierce the cortical bone), scores (elongate indentations that do not pierce the cortical bone), and furrows (elongate indentations that do pierce the cortical bone) (Fig 2). When striated marks, in which the individual denticles of a serrated tooth leave subscores [7], were identified, additional measurements characterizing striation spacing were taken and minimum body mass estimates were generated to help differentiate potential actors [16].

As carnivores are generally inclined to prefer more nutrient-rich muscular tissue or viscera to the bone itself, skeletal elements were categorized according to associated nutritional value within the skeleton. High economy elements are associated with large muscle placement on the body or proximity to viscera, whereas low economy elements are associated with proximity to cartilage and ligaments rather than musculature or viscera. These designations previously have been based on actualistic research on mammalian predators and prey [e.g., 36], but have successfully been applied to both modern [37] and extinct dinosaurian groups [38], with modifications made to account for major differences in prey anatomy. To allow more in depth discussion of the for relative nutritional value of vertebrae, which are generally low economy in mammals, but range into higher nutritional value in dinosaurs, especially in the region of the base of the tail, we have further broken down these elements by anatomical region.

**Fig 3:**
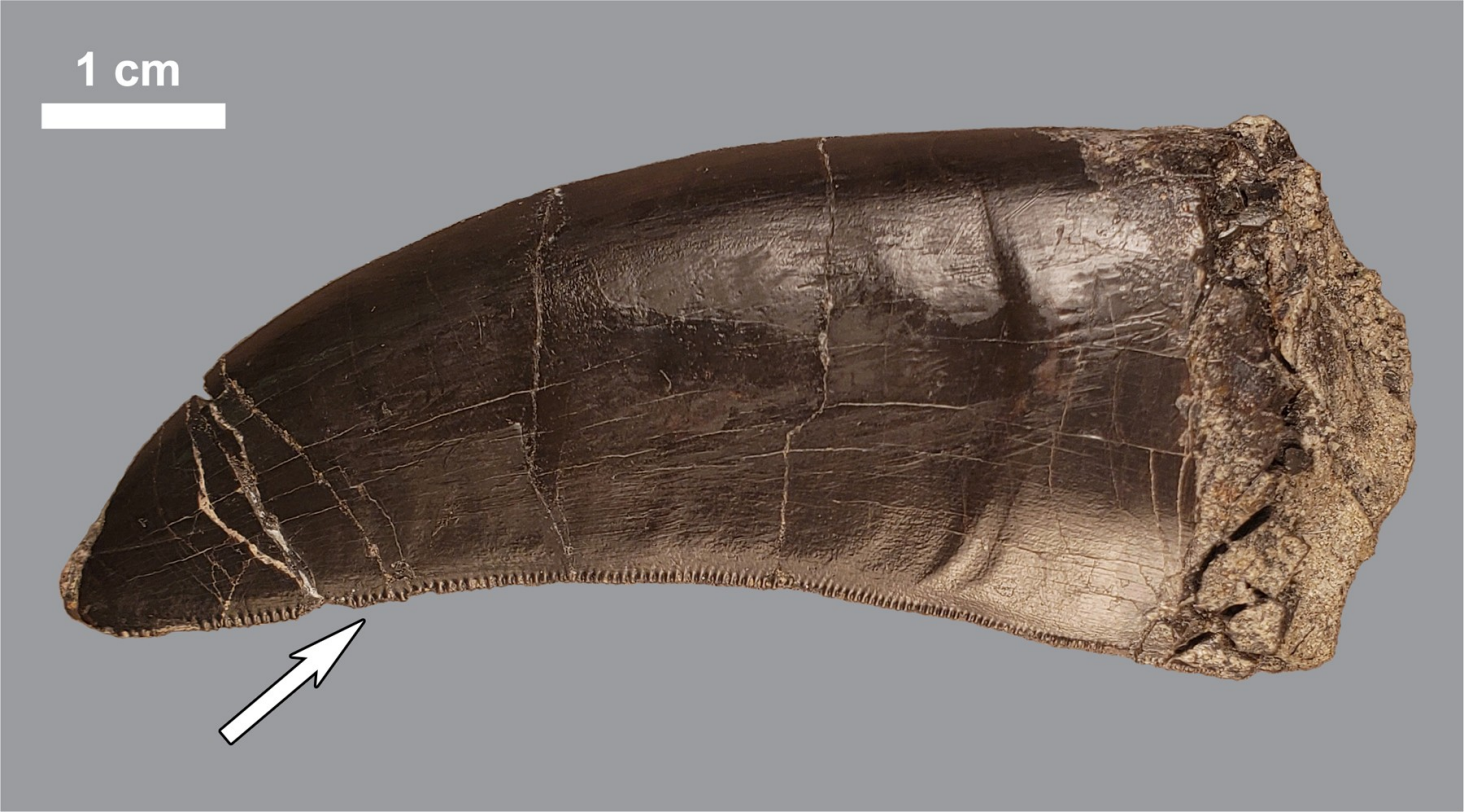
Shed lateral tooth of *Allosaurus* sp. (MWC 5011) found at the Mygatt-Moore Quarry, white arrow indicates the distal denticles. Mesial denticles are present on such teeth, but were not preserved in this specimen.

Ziphodont teeth (Fig 3) can produce striated tooth marks (Fig 2B) on bone surfaces [7, 17, 19, 39–41]. Known as the ichnotaxa *Linichnus serratus* and *Knethichnus parallelum* [42], these striated marks form when the actor’s denticles contact bone surfaces. It has been shown experimentally that the denticle widths may be transcribed as striated marks [16], and several attempts have been made to identify potential ziphodont archosaur actors using them on fossils [40, 41, 43]. We attempted to extrapolate denticle widths from our sample taken from digital photographs. We positioned a Nikon D5300 DSLR camera with an 18–55 mm AF Lens perpendicular to the fossil surface during photography. Using the software ImageJ [44], a line was drawn across the widest part of striation convergence. This line started at one side of the indentation of the first striation and ended at the opposite side of the last. As striations can underestimate, but not overestimate, the size of the denticles that produced them [16], the widest point would have striation widths closest to those of the denticles. As striations were filled with matrix, they were easy to distinguish from the fossil surface. This distance was then divided by the number of striations, giving the average striation width for the mark.

Fossil striation widths were compared to recorded values of denticle widths of genera found at MMQ [45, 46] (S1 Table) to determine the identity and maximum size theropods that could have produced them. This is because denticle widths increase with a theropod’s size [16, 45, 47, 48]. D’Amore & Blumenschine [16] determined this relationship to be negatively allometric (specifically, logarithmic) and may be expressed as a linear relationship between the average denticle width of a given theropod tooth and the natural logarithm of a theropod’s tooth or body size. We therefore used striation widths in these linear equations to extrapolate the maximum length of the tooth at the base (referred to as Crown Base Length [CBL] here, and collected by numerous authors [39, 45, 49–52]), the maximum length of the head, and the body length from head to tail [taken from 53, 54]. As denticles from the mesial and distal carinae differ on average, equations for both carinae were considered as either could have produced the mark. For linear equations, see Table 1 [taken from 16].

**Table 1:** Linear equations used on denticle spacing. The symbol “y” represents the average denticle width of a given theropod tooth for either carina, and “x” represents the natural-logarithm adjusted body size measurement. Striation widths were plugged in as “y” for tooth marked fossils.

Carina	Equation	Measurement
mesial	y = 0.1586x-0.0400	Tooth crown base length (mm)
mesial	y = 0.1725x-0.4588	Skull length (m)
mesial	y = 0.2007x-0.0155	Body length (m)
distal	y = 0.1259x-0.0523	Tooth crown base length (mm)
distal	y = 0.1397x-0.4332	Skull length (m)
distal	y = 0.1642x-0.0689	Body length (m)

## Results

### Bite mark frequency

The survey of the MMQ collection revealed 884 specimens preserving some type of bone surface modification (BSM), with bite marks and insect traces being the most commonly observed, representing 37.331% of specimens examined (Tables 2 and S2). Of these 884, most specimens preserved multiple marks and many preserved multiple types of marks, with bite marks being the most commonly observed BSM (S2 Table). Bite marks were present on 684 specimens (28.926% of surveyed material) and represented 69.893% of all observed BSM (Table 3). Of identified bite marks, individual scores were the most common type of mark, representing 58.216% of the dataset. These numbers are higher than expected, given previous surveys of theropod and other ziphodont taxa’s bite marks [7, 14].

**Table 2:** Examined fossil material from the Mygatt-Moore Quarry.

Taxon	Bite Marked	Total Marked	Unmarked Bones	Total Bones	% Bite Marks	% Total BSM
Sauropoda	436	582	482	1064	40.977%	54.699%
Theropoda	83	105	323	428	19.393%	24.533%
*Mymoorapelta*	26	28	146	174	14.943%	16.092%
Other Tetrapods	84	110	190	300	28.000%	36.667%
Fragment Buckets	56	59	343	402	13.930%	14.677%
**Total**	**685**	**884**	**1484**	**2368**	**28.926%**	**37.331%**

**Table 3:** Types of bone surface modifications found within the Mygatt-Moore assemblage. Numerous elements within the assemblage preserved multiple types of traces. This is a tabulation of all individual traces, not of individual bone elements as in Table 2.

	Theropod Material	Sauropod Material	*Mymoorapelta maysi*	Other Tetrapods	Fragment Buckets	Total Marks	Percent Marked
**Bite Marks**	**260**	**1060**	**31**	**97**	**61**	**1509**	**69.893**
Edge Marks	1	0	0	0	0	1	0.049
Furrows	6	22	0	6	0	34	1.658
Pits	27	40	9	13	1	90	4.388
Serial Pits	5	1	0	0	0	6	0.293
Punctures	12	18	1	3	1	35	1.706
Scores	175	877	20	67	55	1193	58.216
Serial Scores	19	53	0	1	4	77	3.754
Striations/Striated Scores	16	45	1	6	0	68	3.315
Striated Furrows	0	4	0	1	0	5	0.244
**Insect Traces**	**61**	**340**	**5**	**28**	**1**	**435**	**20.148**
Pits/Furrows	61	323	5	28	1	418	20.380
Bore Holes/Chambers	0	12	0	0	0	12	0.585
Bioglyph Scrapes	0	5	0	0	0	5	0.244
**Other Marks**	**24**	**172**	**0**	**12**	**7**	**215**	**9.958**
Abrasion	2	5	0	1	0	8	0.390
Depressions	3	36	0	1	1	41	1.999
Etching	0	4	0	1	0	5	0.244
Fractures	3	3	0	1	0	7	0.341
Prep Damage	5	11	0	5	0	21	1.024
Root Marks	11	108	0	3	6	128	6.241
Other/Unknowns	0	5	0	0	0	5	0.244

When fossil material preserving bite marks was categorized taxonomically, the highest proportion of bite marks were found on sauropod material (70.245%), while theropod material had the second highest proportion of the documented bite marks (17.230%). Other tetrapod taxa, material recovered as small bone fragments (collected in “fragment buckets”), and material identified as belonging to *Mymoorapelta maysi* represented significantly lower portions of the bite mark dataset (Table 3).

Frequencies of bite marks were surveyed from all positively identified skeletal elements (i.e., excluding bone fragments) in each taxonomic group were parsed according to associated nutrient values of a vertebrate carcass. Low economy elements preserve 52.876% of observed bite marks, while high economy elements preserve 47.124%. Among these elements, vertebrae (46.904%) and ribs (31.911%) preserve the majority of bite marks (Table 4).

**Table 4:** Skeletal elements preserving bite marks categorized by associated carcass nutrient availability.

		Theropod Material	Sauropod Material	*M*. *maysi* Material	Other Tetrapods	Total Marks	Percent Marked
**Low Economy Elements**							
	Cervical Centra	1	15	*2*	0	18	7.200%
	Cervical Neural Arches	2	29	0	0	31	12.400%
	Dorsal/Sacral Centra	10	16	4	0	30	12.000%
	Dorsal/Sacral Neural Arches	4	15	0	0	19	7.600%
	Caudal Centra	13	37	2	0	52	20.800%
	Caudal Neural Arches	1	12	1	0	14	5.600%
	Misc. Vertebrae / Fragments	5	76	0	5	86	34.400%
	**Vertebrae Subtotal**	**36**	**200**	**9**	**5**	**250**	**46.904%**
	Haemal Arches	3	17	1	2	23	4.267%
	Tarsals	0	1	0	0	1	0.186%
	Carpals	1	0	0	0	1	0.186%
	Phalanges	4	0	1	0	5	0.928%
	Skull Elements	3	0	0	2	5	0.928%
	**Total**	**47**	**218**	**11**	**9**	**285**	**52.876%**
**High Economy Elements**							
	Ribs	14	108	13	37	172	31.911%
	Pectoral Girdle	0	8	0	0	8	1.484%
	Humeri	1	0	1	0	2	0.371%
	Radii	0	2	0	0	2	0.371%
	Ulnae	0	1	0	0	1	0.186%
	Metacarpals	3	1	0	0	4	0.742%
	Pelvic Girdle	1	13	1	0	15	2.783%
	Femora	0	3	0	0	3	0.557%
	Tibiae	7	2	0	0	9	1.670%
	Fibulae	5	3	0	0	8	1.484%
	Metatarsals	8	2	0	1	11	2.041%
	Limb Fragments	1	17	0	1	19	3.525%
	**Total**	**40**	**160**	**15**	**39**	**254**	**47.124%**

### Identification of the trace maker

While individual tooth mark size ranges greatly throughout the MMQ specimens, the largest bite marks reach 28.26 x 8.16 mm, while the smallest measure 1.49 x 0.19 mm. The larger sizes exclude any small to medium bodied predators in the MMQ ecosystem, leaving crocodyliforms and theropod dinosaurs as the most likely culprits for these larger marks. Smaller marks present more ambiguity, as these data could indicate smaller taxa or juveniles of larger groups as potential actors, or larger individuals’ whose teeth did not make forceful or full contact with the bone.

Crocodyliforms are rare, but present, at the MMQ, which supports the interpretation of a lack of long-term standing water during site formation [30]. Crocodyliform teeth are generally conical with a prominent carina, and while some crocodyliforms deviate from this morphology [55, 56], all taxa known from the MMQ assemblage have these generalized teeth. Bite marks associated with this type of dentition present as round to teardrop-shaped bite marks with a single subscore in the main body of the bite, called a bisection [e.g., 4, 6, 8–10]. The MMQ marks are not round, nor do they exhibit bisections. Instead, they are more fusiform in shape and some have well-defined striations (Fig 2B and 2F), both traits that are associated with the laterally compressed, serrated teeth found in ziphodont dentition [7]. Therefore, in the absence of any known ziphodont crocodyliforms from the MMQ assemblage, this clade can be excluded as the potential trace maker.

The only animals present in the Morrison Formation with ziphodont dentition are theropod dinosaurs. *Allosaurus* is by far the most common theropod genus at the site, but shed teeth of the smaller theropod *Ceratosaurus* are also present, if rare [29, 30, 57]. These two taxa have significant overlap in overall body size across ontogeny, with full-grown *Allosaurus* reaching a larger known maximum body length (approximately 8.5 meters) than *Ceratosaurus* (over 6.2 meters) [53, 54]. These species also have overlapping values concerning both mesial and distal average denticle widths [45, 46]. Measurements based on tooth mark spacing [12] and striation width [16] provide the means for estimating body sizes. However, biting events in which the individual teeth are not moving perpendicular to the acting section of the tooth row can result in both serial bite marks that appear more closely spaced than the initiating teeth actually were [12] and individual striations that are spaced more closely than their corresponding denticles of the acting teeth [16]. Therefore, estimates generated from these measurements should be considered a lower bound for potential body sizes of the trace makers.

Six striated marks with clear, visible striations were measured to determine average striation widths (Table 5). The number of parallel striations ranged from 3–11, and the width of the mark ranged from 0.61–4.20 mm. The average striation widths for each of these marks ranged from 0.204–0.651 mm. Five of the six marks have average striation widths that fall either within or below the typical denticle widths of contemporaneous large theropods recorded in the literature found at the MMQ, specifically members of *Allosaurus* and *Ceratosaurus* [45, 46] (S1 Table). Two of the larger marks correlate to denticle width ranges restricted to premaxillary teeth for both taxa, as well as a single first maxillary tooth of *Ceratosaurus*, for the distal carinae (MWC 3763 and MWC 2730). The mark with the largest striation width found on the dorsal surface of a theropod pedal claw (MWC 7263; Fig 2A) suggests denticle widths larger than any known taxon from the MMQ, but has been found in larger, non-contemporaneous taxa like *Tyrannosaurus rex* [45]. This measurement falls only slightly above the average denticle width of the contemporaneous *Torvosaurus tanneri* [58]. Hendrickx and Mateus [59] reported an average of 8 denticles per 5 mm (or 0.625 mm average denticle width using our metric) in both the European and North American *Torvosaurus* species.

**Table 5:** Actor body size estimates based on denticle spacing.

Specimen	Striation Width (mm)	Number of Striations	Mark Maker Carina	Measurement	Size
MWC 8675	0.2043	3	mesial	Tooth crown basal length (mm)	4.67
*Allosaurus sp*.			mesial	Skull length (m)	0.23
dorsal centrum			mesial	Body length (m)	2.56
			distal	Tooth crown basal length (mm)	3.34
			distal	Skull length (m)	0.19
			distal	Body length (m)	2.28
MWC 3763	0.4894	7	mesial	Tooth crown basal length (mm)	28.17
*Mymoorapelta maysi*			mesial	Skull length (m)	1.19
dorsal rib			mesial	Body length (m)	10.61
			distal	Tooth crown basal length (mm)	32.21
			distal	Skull length (m)	1.50
			distal	Body length (m)	12.95
MWC 7263	0.3048	3	mesial	Tooth crown basal length (mm)	8.79
Theropoda			mesial	Skull length (m)	0.41
pedal claw			mesial	Body length (m)	4.23
			distal	Tooth crown basal length (mm)	7.43
			distal	Skull length (m)	0.40
			distal	Body length (m)	4.21
MWC 2730	0.4991	7	mesial	Tooth crown basal length (mm)	29.93
*Allosaurus sp*.			mesial	Skull length (m)	1.26
caudal vertebra			mesial	Body length (m)	11.13
			distal	Tooth crown basal length (mm)	34.77
			distal	Skull length (m)	1.60
			distal	Body length (m)	13.73
MWC 9407	0.3905	11	mesial	Tooth crown basal length (mm)	15.10
*Allosaurus sp*.			mesial	Skull length (m)	0.67
caudal centrum			mesial	Body length (m)	6.48
			distal	Tooth crown basal length (mm)	14.68
			distal	Skull length (m)	0.74
			distal	Body length (m)	7.09
MWC 7263	0.6505	4	mesial	Tooth crown basal length (mm)	77.78
Theropoda			mesial	Skull length (m)	3.04
pedal claw			mesial	Body length (m)	23.67
			distal	Tooth crown basal length (mm)	115.76
			distal	Skull length (m)	4.74
			distal	Body length (m)	34.54

When striation widths were used to extrapolate tooth and body sizes, a wide range of values resulted (Table 5). Four of the six extrapolated CBL measurements fell within the typical tooth size ranges of *Allosaurus*. The results were similar concerning *Ceratosaurus*, except one of these marks (MWC 2730), when predicted to be produced by the mesial carina, yielded a tooth size larger than the largest maxillary teeth of *Ceratosaurus* recorded (UMNH VP5278 maxillary tooth 5, [45, 60]. The mark with the largest striation widths (MWC 7263) yielded a much larger CBL than any MMQ theropod on record. Extrapolated skull and body lengths ranged from much smaller, to much larger, than any *Allosaurus* or *Ceratosaurus* (and, for the largest, any theropod) recorded. Many of the striations that yielded CBLs that align well with these taxa also yielded head and body sizes that were unrealistically large for them. Some of these extrapolations do coincide with those predicted for the large theropod *Saurophaganax maximus* (OMNH 01123), which is not present in the MMQ assemblage but is known from the Morrison Formation of western Oklahoma. *Saurophaganax maximus* is a gigantic theropod estimated to be 25% bigger than the largest known *Allosaurus* specimens. Although the taxonomic identity of OMNH 01123 has been debated as either an exceptionally large *Allosaurus* or a separate taxon [53, 61, 62], its size is generally agreed upon. *Torvosaurus tanneri*, with a body length of up to 10m [58], fell just below the extrapolated body sizes based on two of the larger marks (MWC 3763 and MWC 2730) when assumed to be produced by the mesial carinae.

## Discussion

### Bite mark frequency

In previous research on theropod modified assemblages, bite marks have often been determined to be extremely rare [13, 14](. A large survey of bite mark frequencies in dinosaurian and mammalian dominated assemblages, which included four Morrison Formation sites, determined that dinosaurs and mammals utilize prey bone in fundamentally different ways. Among the mammalian-modified assemblages, between 13.1 and 37.5% of all bones preserved at least one type of bite mark, while the dinosaurian-dominated assemblages yielded bite marked bone frequencies between 0 and 4%. These differences were used to interpret that dinosaurian and mammalian feeding strategies were fundamentally different, with mammals specifically targeting bone as a food resource and dinosaurs actively avoiding it [14].

The results of the MMQ bite mark survey present a stark contrast with these previous studies. With 28.927% of all observed bones exhibiting at least one bite mark, the MMQ assemblage falls well above the predicted range of modifications from other (especially non-tyrannosaur) dinosaur-modified assemblages, and in fact is positioned solidly in the more ‘mammalian’ range of modification frequencies [14]. It is unclear whether this higher rate of bite marked bones represents something unique about local trophic dynamics or preservational history of the MMQ, or whether the inflated counts are caused by more complete collection protocols used by the MWC in comparison to other Morrison Formation sites.

If the MMQ bite mark frequencies represent a true deviation from the norm, then why were the theropods in this paleoecosystem more destructive of bone than in other assemblages, even ones from other Morrison Formation sites? Carnivore diversity and feeding behavior can affect element modification and survival, especially if frequencies of osteophagous taxa [e.g., 20] change in an ecosystem [63]. However, the taxonomic composition of the MMQ assemblage does not differ substantially from other Morrison sites, though species richness is generally lower [30], and no truly osteophagous taxa [i.e., 20] are known from the formation. Higher numbers of carnivores interacting with a single set of remains, either because of social behavior or scavenging succession, can result in more complete and rapid processing of remains [64]. The slow deposition of laminated shales in the MMQ environment would have promoted long exposure times for remains, particularly among large animals like sauropod dinosaurs. In times when other easy sources of food were not readily available (i.e., dry seasons), this would expose skeletons to prolonged, more complete scavenging that might otherwise be expected. This suggests that the MMQ might preserve a stressed paleoecosystem, in which any available remains would be more fully processed to ensure utilization of every available nutrient source.

However, the heightened bite mark frequencies found in the MMQ might not reflect a biological signal at all, and may instead be the result of collector bias. In 2016, the MWC shifted from collecting only specimens deemed to be of a sufficient quality to bulk collection of all fossils found at the MMQ. Bulk collection is rarely used in vertebrate paleontology, as it can be cumbersome for both field crews and repositories. However, preliminary work at the MMQ on the bulk collected material does seem to indicate that collection protocols based on perceived ‘value’ or ‘attractiveness’ is biased against remains with bone surface modifications [65]. This has significant potential for skewing paleoecological analyses based on these surficial traces.

### Identification of the trace maker

Because most striation widths fell into the range of denticle widths of both large theropods known from the MMQ, we can reliably suggest that at least some of these traces were made by grown *Allosaurus* or *Ceratosaurus*. The large theropod *Saurophaganax maximus* (OMNH 01123) is known only from the Morrison Formation of Oklahoma and could have also produced said marks. Another possible candidate is *Torvosaurus tanneri* from the Morrison Formation in Colorado, Utah, and Wyoming. Neither of these have been identified from skeletal remains in the MMQ assemblage, but the largest set of bite mark striations recorded in this study are consistent with theropods of their size, and could not have been produced by *Allosaurus* or *Ceratosaurus* based on our present knowledge of them. There are no records of denticle widths of *Saurophaganax* for direct comparison, but extrapolated CBLs suggest an animal of its size could be the culprit. *Torvosaurus* had average denticle widths only slightly below the largest striation widths, and, because these measurements are in fact averages, it is very possible that contact between a maxillary tooth’s larger denticles could have produced the largest striation widths seen here. Therefore, the largest striations are consistent with either an *Allosaurus* larger than any known specimen or a separate taxon (such as *Saurophaganax* or *Torvosaurus*) not previously reported from the MMQ. This result is particularly interesting because it either increases the known diversity of the site based on ichnological evidence alone, or represents powerful evidence of cannibalism in *Allosaurus*.

As for the identity of the trace maker responsible for the more closely spaced striations, striation widths can underestimate actual denticle widths [16]. Therefore, it is unclear if the marks with smaller striation widths were produced by smaller actors or the same large theropods. Nevertheless, large theropods including *Allosaurus*, *Ceratosaurus*, *Torvosaurus*, and the OMNH 01123 theropod remain the only possible actors that we know of that could have produced the marks with the larger striation widths. The fact that two of the six striated marks correlate well to premaxillary teeth in *Allosaurus* and *Ceratosaurus* is not surprising, as these teeth have been postulated to be used for defleshing carcasses in large theropods in the past [12].

This study shows that applying striated tooth marks to predictive equations of the characteristics of actors may result in varied effectiveness. Striation widths all yielded tooth CBLs within the ranges of contemporaneous archosaurs, but skull and body lengths were widely distributed. Several factors may have influenced the latter extrapolations and negatively affected their reliability, and these were previously addressed by D’Amore and Blumenschine [16]. There typically exists a range of denticle widths for different teeth along the arcade, as size heterodonty is apparent in theropods [66] and denticle size is correlated with said tooth size [48]. A tooth mark with an accurate transcription of denticle widths from a tooth with very large or small denticles for the individual would misrepresent the skull and body size. Heterodonty in tooth and denticle size appears also to increase with overall body size, making this more likely in larger theropods. In addition, the logarithmic nature of these equations results in less separation between larger theropod individuals. This is noted by Chandler [47], who stated that *Allosaurus* and *Tyrannosaurus* denticle widths were not significantly different regardless of the dramatic differences in both crown and body size characters. Therefore, slight variability in striation widths results in large variations in correlating size characters. As we have shown in practice here, this methodology is well suited to for establishing whether or not a large actor created the mark and less reliable for deriving morphological data about said large actor.

### Behavior of the trace maker

In general, predators will take advantage of the most easily attained food resources available to them, and scavenging represents, in essence, an opportunity for a free meal (in terms of energy expenditures). In nutrient poor environments, more common and complete scavenging can become a critical source of nutrients for carnivores and a more common cause of bone surface modifications [63]. Taphonomic reconstructions of MMQ site formation suggest a riparian system with slow sediment accumulation, resulting in long exposure times for skeletons [65]. Longer residence time leaves remains vulnerable to alteration by different biotic and abiotic taphonomic processes, including trampling, insect burrowing, abrasion, weathering, and most important to this study, scavenging [e.g., 67, 68].

Differentiating bite marks generated by predation versus those created by scavenging events can be challenging, with most arguments supporting identification of scavenging relying on size comparisons between predator and prey, in which scavengers are essentially documented feeding unexpectedly far above their preferred prey weight [69, 70, 71], or on discussions of mark location, site taphonomy, or relative prey element economy [36, 38, 72, 73]. In these analyses, regions of the prey taxon’s anatomy are parsed by perceived nutritional value. Some regions of the vertebrate skeleton have a higher nutrient value related to associated soft tissues, and are therefore targeted first, while others are of less nutritional value and are therefore targeted last. This results in a predictable pattern of consumption known as the scavenging sequence, best documented among mammalians [37, 74–76], but broadly applicable to other vertebrate groups as well [38]. Bite marks on high economy bones are therefore associated with predation [e.g., 4], or at least early access to remains, while feeding traces on only low economy bones are interpreted to be caused by late access to remains, such as scavenging [e.g., 38].

Among the bite marks identified in this study, patterns of bite mark location vary based on the affected taxon. Among the sauropods and ornithischians, 43.317% of observed bite marks are found on high economy regions of the skeleton (Table 4), such as long bones, targeted alongside the high nutrient musculature they support, and ribs. Concerning mammals these are often modified in early stage feeding when the animal’s viscera is targeted [e.g., 36, 37, 74–76], and it is reasonable to assume theropods would do the same. These feeding traces are most consistent with early access to remains, or predation. The remaining 56.683% are on low economy elements, such as phalanges, vertebrae, and haemal arches, suggesting these elements were either late access remains or scavenged.

By comparison, 54.023% of the modified theropod skeletal elements are lower economy elements, while 45.977% are found on higher economy bones (Table 4). However, the possible association of these bite marks with conspecifics (i.e., possible *Allosaurus* bite marks on *Allosaurus* remains) suggests that interpretations other than feeding might be responsible for these modifications. Are these traces not related to feeding at all, and are instead represent evidence of inter- or intraspecific competition? Crocodyliforms, both extant and extinct, provide some basis of comparison for fighting behavior among large-bodied, non-avian archosaurs [e.g., 77–79]. Members of this clade often target their opponent’s head, base of the tail, and limbs near major joints such as the hip or knee (i.e., grasping sites after Njau and Blumenschine, [6]. Fights of this nature are not always fatal, and a large proportion of individuals are expected to retain healed evidence of such fights [77]. When an opponent is killed, the line between intraspecific competition and feeding is blurred, when defeated opponents subsequently provide a convenient meal.

In the MMQ bite marks, none of the observed traces preserve evidence of remodeling or reaction tissue [e.g., 80, 81], suggesting that whatever the source of the bites, none of the individuals survived the incidents long enough to heal. Additionally, the bite marks identified on *Allosaurus* distal limb elements in this study are not consistent with comparable behaviors among extant analogues, and some, especially those on the centra of trunk vertebrae and deeply buried regions of the haemal arches, could only reasonably be reached for modification after death and significant dismemberment [81]. Therefore, we reject inter- or intraspecific competition as a viable hypothesis for all of the bite marks observed and instead interpret them as feeding traces.

**Fig 4:**
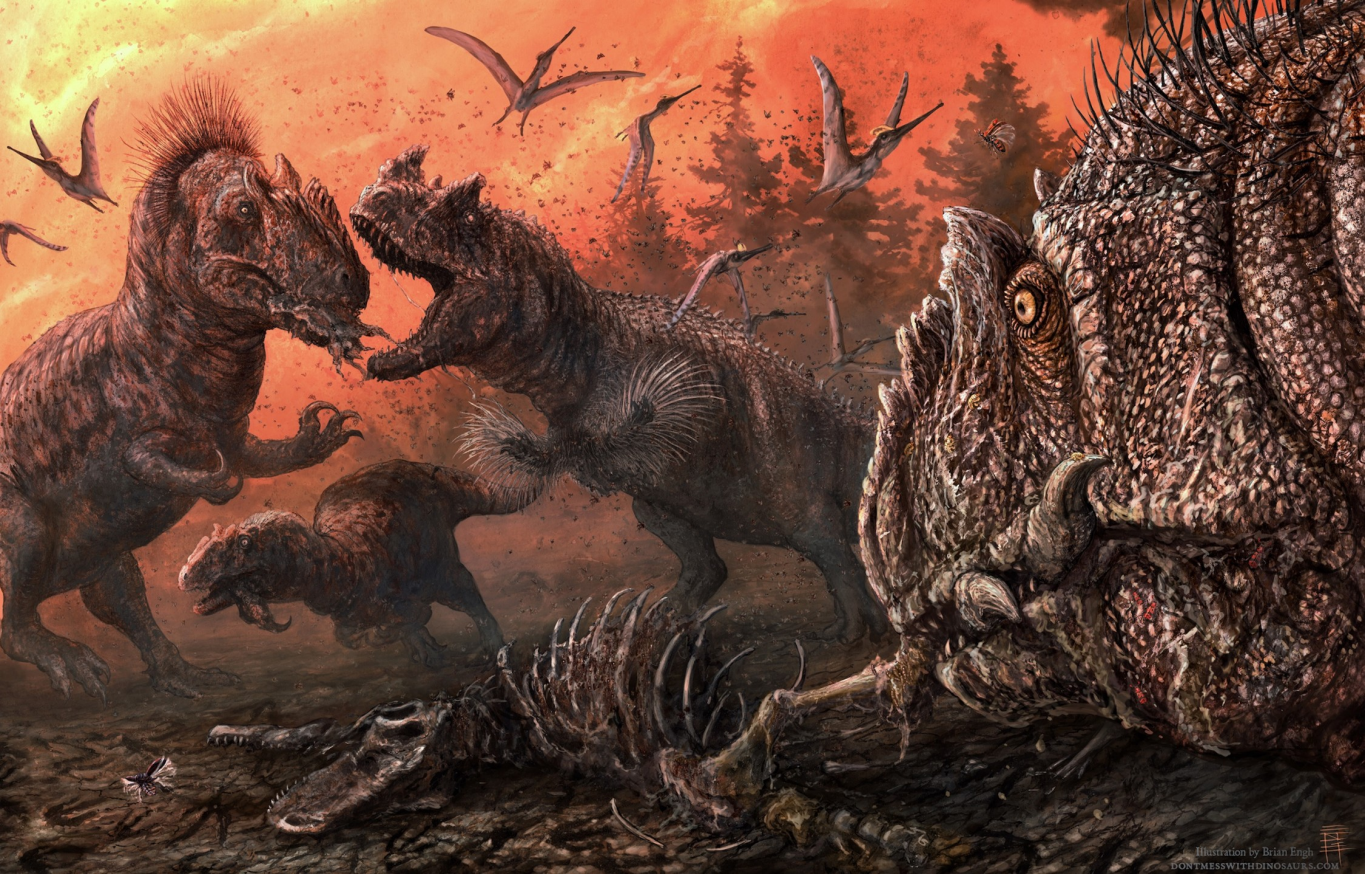
Dry season at the Mygatt-Moore Quarry showing *Ceratosaurus* and *Allosaurus* fighting over the desiccated carcass of another theropod. Illustration by Brian Engh (dontmesswithdinosaurs.com).

Scavenging between large carnivores, including cannibalism, is fairly common among modern groups [e.g., 82–84], but direct evidence for it in the fossil record is extremely rare. Most cases of cannibalism among theropods has only been tentatively suggested [85, 86]. Definitive evidence through striated tooth marks has been recorded only in *Tyrannosaurus rex* [38] and *Majungasaurus crenatissimus* [40], but never before in *Allosaurus* or *Ceratosaurus*. Given the relative abundances of the theropods known from the MMQ [30], it is the most parsimonious interpretation that many of the bite marks reported here may represent the first known example of cannibalism in *Allosaurus* (Fig 4)

## Conclusions

The Mygatt-Moore Quarry preserves an unusually highly tooth-marked assemblage from the Upper Jurassic Morrison Formation. Bite marks are consistent with a theropod trace maker, and striations place the traces within the range expected for the known large-bodied theropods from the site: *Allosaurus* and *Ceratosaurus*. The largest of these traces suggests an individual that is too large to be either taxon based on existing fossils, suggesting they were produced by an even larger taxon such as *Saurophaganax* or *Torvosaurus*. While the location of traces on herbivorous dinosaurs are consistent with predation or early access to remains, bite marks found on other theropod material, more specifically *Allosaurus*, are concentrated on lower-economy bones, suggesting that they represent incidences of scavenging. If the trace maker is *Ceratosaurus*, this study represents the first incidence of this taxon feeding on another large, contemporaneous theropod. If the trace maker is *Allosaurus*, this study represents the first time cannibalism has been reported in this taxon and its encompassing clade, Allosauroidea. If the trace maker is a taxon not represented in the fossil assemblage (i.e., *Saurophaganax* or *Torvosaurus*), then these bite marks preserve the first indirect evidence of such a taxon in the MMQ, raising the diversity of large carnivores at the site based on bone surface modifications alone in the absence of body fossils. This seems likely for our largest striations, as they are too large to be produced by any taxon of known size in the MMQ.

Together with the high volume of other bone surface modifications, these traces suggest a depositional environment in which remains were exposed at the surface for long stretches of time, allowing more complete utilization of decaying remains than might be expected at other, contemporary sites with more rapid sediment accumulation (e.g., Carnegie Quarry-Dinosaur National Monument). Therefore, the high concentration of bone surface modifications at the MMQ may represent a true sampling of the processes that shaped the fossil site, a signal that seems to have been boosted by a recent shift to bulk collection at the locality. More detailed comparisons of bone surface modification frequencies in samples collected both before and after this change in collection protocol is ongoing, but this case study demonstrates that paleoecological analyses of these taphonomic processes are helped by more complete sampling and are actively biased by targeting of less damaged, more aesthetically-pleasing bones, as is common practice when type and exhibition specimens are preferentially collected.

## Supporting information

S1 Table (XLSX)

S2 Table (XLSX)

S3 Table (XLSX)

## References

[1] Boyd CA, Drumheller SK, Gates TA. 2013. Crocodyliform feeding traces on juvenile ornithischian dinosaurs from the Upper Cretaceous (Campanian) Kaiparowits Formation, Utah. PloS ONE. 2013;8(2): e57605 10.1371/journal.pone.0057605 23460882 PMC3584051

[2] Noto CR, Main DJ, Drumheller SK. Feeding traces and paleobiology of a Cretaceous (Cenomanian) crocodyliform: example from the Woodbine Formation of Texas. Palaios. 2012;27(2): 105–115.

[3] DePalma RA, Burnham DA, Martin LD, Rothschild BM, Larson PL. Physical evidence of predatory behavior in Tyrannosaurus rex. P Natl A Sci. 2013;110(31): 12560–12564. 10.1073/pnas.1216534110 PMC3732924 23858435

[4] Drumheller SK, Brochu CA. A diagnosis of Alligator mississippiensis bite marks with comparisons to existing crocodylian datasets. Ichnos. 2014;21: 131–146.

[5] Labandeira CC. A paleobiologic perspective on plant–insect interactions. Curr Opin Plant Biol. 2013;16(4): 414–421. 10.1016/j.pbi.2013.06.003 23829938

[6] Njau JK, Blumenschine RJ. 2006. A diagnosis of crocodile feeding traces on larger mammal bones, with fossil examples from the Plio-Pleistocene Olduvai basin, Tanzania. J Hum Evol. 2006;50: 142–162. 10.1016/j.jhevol.2005.08.008 16263152

[7] D’Amore DC, Blumenschine RJ. Komodo monitor (Varanus komodoensis) feeding behavior and dental function reflected through tooth marks on bone surfaces, and the application to ziphodont paleobiology. Paleobiology. 2009;35: 525–552.

[8] Drumheller SK, Brochu CA. Phylogenetic taphonomy: a statistical and phylogenetic approach for exploring taphonomic patterns in the fossil record using crocodylians. Palaios. 2016;31(10): 463–478.

[9] Njau J, Gilbert H. Standardizing terms for crocodile-induced bite marks on bone surfaces in light of the frequent bone modification equifinality found to result from crocodile feeding behavior, stone tool modification, and trampling. FOROST (Forensic Osteology) O P. 2016;3: 1–13.

[10] Drumheller SK, D’Amore DC, Njau JK. Taphonomic approaches to bite mark analyses in the fossil record and applications to crocodyliform and broader archosaurian paleobiology In: Woodward-Ballard HN, Farlow JO, editors. Crocodylian Biology and Paleobiology. Bloomington, IN: Indiana University Press; Forthcoming.

[11] Pobiner B. Paleoecological information in predator tooth marks. J Taphonomy. 2008;6(3–4): 373–397.

[12] Hone DW, Chure DJ. Difficulties in assigning trace makers from theropodan bite marks: an example from a young diplodocoid sauropod. Lethaia. 2018;51(3): 456–466.

[13] Carpenter K. Evidence of predatory behavior by carnivorous dinosaurs. Gaia. 1998;15: 135–144.

[14] Fiorillo AR. Prey bone utilization by predatory dinosaurs. Palaeogeogr Palaeocl Palaeoeco. 1991;88: 157–166.

[15] Jacobsen AR. 2001 Tooth-marked small theropod bone: an extremely rare trace In: Tanke DH, Carpenter K, editors. Mesozoic Vertebrate Life: New Research Inspired by the Research of Philip J. Currie; Bloomington: Indiana University Press; pp. 58–63.

[16] D’Amore DC, Blumenschine RJ. Using striated tooth marks on bone to predict body size in theropod dinosaurs: a model based on feeding observations of Varanus komodoensis, the Komodo monitor. Paleobiology. 2012;38: 79–100.

[17] Erickson GM, Olson KH. Bite marks attributable to Tyrannosaurus rex: preliminary description and implications. J Vertebr Paleontol. 1996;16: 175–178.

[18] Chin K, Tokaryk TT, Erickson GM, and Calk LC. A king-sized theropod coprolite. Nature. 1998;393: 680–682.

[19] Jacobsen AR. Feeding behavior of carnivorous dinosaurs as determined by tooth marks on dinosaur bones. Hist Biol. 1998;13: 17–26.

[20] Gignac PM, Erickson GM. The biomechanics behind extreme osteophagy in Tyrannosaurus rex. Sci Rep-UK. 2017;7(1): 2017. 10.1038/s41598-017-02161-w PMC5435714 28515439

[21] Hone DWE, Rauhut OWM. Feeding behaviour and bone utilisation by theropod dinosaurs. Lethaia. 2010;43: 232–244.

[22] Matthew WD. Allosaurus, a carnivorous dinosaur, and its prey. Am Mus J. 1908;8: 3–5.

[23] Hunt AP, Meyer CA, Lockley MG, Lucas SG. Archaeology, toothmarks and sauropod dinosaur taphonomy. Gaia. 1994;10: 225–231.

[24] Chure DJ, Fiorillo AR, Jacobsen R. Prey bone utilization by predatory dinosaurs in the Late Jurassic of North America, with comments on prey bone use by dinosaurs throughout the Mesozoic. Gaia. 2000;15: 227–232.

[25] Carpenter K, Sanders F, McWhinney L, Wood L. Evidence for predator–prey relationships: example for Allosaurus and Stegosaurus In Carpenter K, editor. The Carnivorous Dinosaurs. Bloomingston IN: Indiana University Press; 2005 pp. 325–350.

[26] Kirkland JI. Morrison fishes. Mod Geol. 1998;22(1): 1–4.

[27] Kirkland JI, Carpenter K. North America’s first pre-Cretaceous ankylosaur (Dinosauria) from the Upper Jurassic Morrison Formation of western Colorado. Brigham Young Univ Geol Stud. 1994;40: 25–42.

[28] Foster JR. Paleoecological analysis of the vertebrate fauna of the Morrison Formation (Upper Jurassic), Rocky Mountain Region, USA. New Mex Mus Nat Hist Sci Bull. 2003;23 1–95.

[29] Foster J. Paleontology, taphonomy, and sedimentology of the Mygatt-Moore Quarry, a large Dinosaur Bonebed in the Morrison Formation, Western Colorado: Implications for Upper Jurassic Dinosaur Preservation Modes. BLM Report. 2014: pp. 70.

[30] Foster JR, Hunt-Foster RK, Gorman MA II, Trujillo KC, Suarez CA, McHugh JB, et al Paleontology, taphonomy, and sedimentology of the Mygatt-Moore Quarry, a large dinosaur bonebed in the Morrison Formation, western Colorado: implications for Upper Jurassic dinosaur preservation modes. Geol Intermountain West. 2018;5: 23–93.

[31] Foster JR, Hunt-Foster RK. New occurrences of dinosaur skin of two types (Sauropoda? and Dinosauria indet.) from the Late Jurassic of North America (Mygatt-Moore Quarry, Morrison Formation). J Vertebr Paleontol. 2011;31(3): 717–721.

[32] Trujillo KC, Foster JR, Hunt-Foster RK, Chamberlain KR. A U/Pb age for the Mygatt-Moore Quarry, Upper Jurassic Morrison Formation, Mesa County, Colorado. Volumina Jurassica. 2014;12(2): 107–114.

[33] McHugh JB. Evidence for niche partitioning among ground-height browsing sauropods during the Late Jurassic Period of North America. Geol Intermountain West. 2018;5: 95–103.

[34] Blumenschine RJ, Marean CW, Capaldo SD. Blind tests of inter-analyst correspondence and accuracy in the identification of cat marks, percussion marks, and carnivore tooth marks on bone surfaces. J Archeol Sci. 1996;23: 493–507.

[35] Binford LR. Bones: ancient men and modern myths. New York: Academic Press; 1981.

[36] Shipman P. Scavenging or hunting in early hominids: theoretical framework and tests. Am Anthropol. 1986;88(1): 27–43.

[37] Behrensmeyer AK, Stayton CT, Chapman RE. Taphonomy and ecology of modern avifaunal remains from Amboseli Park, Kenya. Paleobiology. 2003;29(1): 52–70.

[38] Longrich NR, Horner JR, Erickson GM, Currie PJ. Cannibalism in Tyrannosaurus rex. PloS One. 2010;5(10): e13419 10.1371/journal.pone.0013419 20976177 PMC2955550

[39] Currie PJ, Rigby JK Jr, Sloan RE. Theropod teeth from the Judith River Formation of southern Alberta, Canada In: Carpenter K, Currie PJ, editors. Dinosaur systematics: perspectives and approaches. Cambridge: Cambridge University Press; 1990 pp. 107–125.

[40] Rogers RR, Krause DW, Rogers KC. Cannibalism in the Madagascan dinosaur *Majungatholus atopus*. Nature. 2003;422: 515–518. 10.1038/nature01532 12673249

[41] McLain MA, Nelsen D, Snyder K, Griffin CT, Siviero B, Brand LR, et al Tyrannosaur cannibalism: a case of a tooth-traced tyrannosaurid bone in the Lance Formation (Maastrichtian), Wyoming. Palaios. 2018;33(4): 164–173.

[42] Jacobsen AR, Bromley RG. New ichnotaxa based on tooth impressions on dinosaur and whale bones. Geol Q. 2009;53: 373–382.

[43] Schoch RR, Seegis D. A Middle Triassic palaeontological gold mine: the vertebrate deposits of Vellberg (Germany). Palaeogeogr Palaeocl Palaeoeco. 2016;459: 249–267.

[44] Schneider CA, Rasband WS, Eliceiri KW. NIH Image to ImageJ: 25 years of image analysis. Nat Methods. 2012;9(7): 671–675. 10.1038/nmeth.2089 22930834 PMC5554542

[45] Smith JB, Vann DR, Dodson P. Dental morphology and variation in theropod dinosaurs: implications for the taxonomic identification of isolated teeth. Anat Rec. 2005;285: 699–736. 10.1002/ar.a.20206 15986487

[46] Hendrickx C, Mateus O, Araújo R. A proposed terminology of theropod teeth (Dinosauria, Saurischia). J Vertebr Paleontol. 2015;35(5): e982797.

[47] Chandler CL. Taxonomic and functional significance of serrated tooth morphology in theropod dinosaurs. M.Sc. Thesis, Yale University, 1990.

[48] Farlow JO, Brinkman DL, Abler WL, Currie PJ. Size, shape and serration density of theropod dinosaur lateral teeth. Mod Geol. 1991;16: 161–198.

[49] Holtz TR Jr, Brinkman DL, Chandler CL. Denticle morphometrics and a possible omnivorous feeding habit for the theropod dinosaur Troodon. Gaia. 1998;15: 159–166.

[50] Sankey JT, Brinkman DB, Guenther M, Currie PJ. Small theropod and bird teeth from the Late Cretaceous (Late Campanian) Judith River Group, Alberta. J Paleontol. 2002;76: 751–763.

[51] Larson DW, Currie PJ. Multivariate analyses of small theropod dinosaur teeth and implications for paleoecological turnover through time. PLoS One. 2013;8(1): e54329 10.1371/journal.pone.0054329 23372708 PMC3553132

[52] Torices A, Reichel M, Currie PJ. Multivariate analysis of isolated tyrannosaurid teeth from the Danek Bonebed, Horseshoe Canyon Formation, Alberta, Canada. Can J Earth Sci. 2014;51(11): 1045–1051.

[53] Paul GS. Predatory dinosaurs of the world. New York: Simon and Schuster; 1988.

[54] Therrien F, Henderson DM. My theropod is bigger than yours… or not: estimating body size from skull length in theropods. J Vertebr Paleontol. 2007;27: 108–115.

[55] Brochu CA. Crocodylian snouts in space and time: phylogenetic approaches toward adaptive radiation. Am Zool. 2001;41(3): 564–585.

[56] D’Amore DC, Harmon M, Drumheller SK, Testin JJ. Quantitative heterodonty in Crocodylia: assessing size and shape across modern and extinct taxa. PeerJ. 2019;7: e6485 10.7717/peerj.6485 30842900 PMC6397764

[57] Foster JR, McHugh JB, Peterson JE, Leschin MF. Major bonebeds in mudrocks of the Morrison Formation (Upper Jurassic), northern Colorado Plateau of Utah and Colorado. Geol Intermountain West. 2016;3: 33–66.

[58] Galton PM, Jensen JA. A new large theropod dinosaur from the Upper Jurassic of Colorado. Brigham Young University Geology Studies 1979;26(1):1–12.

[59] Hendrickx C, Mateus O. Torvosaurus gurneyi n. sp., the largest terrestrial predator from Europe, and a proposed terminology of the maxilla anatomy in nonavian theropods. PLoS ONE. 2014;9(3),e88905 10.1371/journal.pone.0088905 24598585 PMC3943790

[60] Madsen JH, Welles SP. Ceratosaurus (Dinosauria, Theropoda): a revised osteology. Utah Geological Survey; 2000.

[61] Hunt AP, Lucas SG. JW Stovall and the Mesozoic of the Cimarron Valley, Oklahoma and New Mexico. New Mex Geol Soc Guidebook. 1987;38: 139–151.

[62] Chure DJ. A reassessment of the gigantic theropod Saurophagus maximus from the Morrison Formation (Upper Jurassic) of Oklahoma, USA. In: Sixth Symposium on Mesozoic Terrestrial Ecosystems and Biota. 1995;6: 103–106.

[63] Faith JT, Behrensmeyer AK. Changing patterns of carnivore modification in a landscape bone assemblage, Amboseli Park, Kenya. J Archaeol Sci. 2006;33(12): 1718–1733.

[64] Willey P, Snyder LM. Canid modifications of human remains: implications for time-since-death estimations. J For Sci. 1989;34(4): 894–901. 2760591

[65] McHugh JB, Drumheller SK, Riedel A, Kane M. An altered assemblage: bone surface modifications on vertebrate material from the Upper Jurassic Mygatt-Moore Quarry in Rabbit Valley, Colorado. Soc Vertebr Paleontol Ann Meeting Prog Abstracts. 2018: 179.

[66] Smith JB. Heterodonty in Tyrannosaurus rex: implications for the taxonomic and systematic utility of theropod dentitions. J Vertebr Paleontol. 2005;25: 865–887.

[67] Behrensmeyer AK. Taphonomic and ecologic information from bone weathering. Paleobiology. 1978;4(2): 150–162.

[68] Lyman RL. Vertebrate Taphonomy. Cambridge: Cambridge University Press; 1994.

[69] Antunes MT. 2017. Huge Miocene crocodilians from Western Europe: predation, comparisons with the “false gharial" and size. Anu I Geociências. 2017;40(3): 117–130.

[70] Drumheller SK, Wilberg EW. A synthetic approach for assessing the interplay of form and function in the crocodyliform snout. Zool J Linn Soc-Lond. 2019; zlz081. 10.1093/zoolinnean/zlz081

[71] Drumheller SK, Stocker MR, Nesbitt SJ. Direct evidence of trophic interactions among apex predators in the Late Triassic of western North America. Naturwissenschaften. 2014;101(11): 975–987. 10.1007/s00114-014-1238-3 25228348

[72] Jennings DS, Hasiotis ST. Taphonomic analysis of a dinosaur feeding site using geographic information systems (GIS), Morrison Formation, southern Bighorn Basin, Wyoming, USA. Palaios. 2006;21: 480–492.

[73] Hone DWE, Watabe M. New information on the feeding behaviour of tyrannosaurs. Acta Palaeontol Pol. 2010;55: 627–634.

[74] Hill AP. Early postmortem damage to the remains of some contemporary East African mammals In: Behrensmeyer AK, Hill AP, editors. Fossils in the Making: Vertebrate Taphonomy and Paleoecology; Chicago: The University of Chicago Press; 1980 pp. 131–155.

[75] Blumenschine R. Carcass consumption sequences and the archaeological distinction of scavenging and hunting. J Hum Evol. 1986;15: 639–659.

[76] Haglund WD. Dogs and Coyotes: Postmortem Involvement with Human Remains In: Haglund WD, Sorg MH, editors. Forensic Taphonomy: The Postmortem Fate of Human Remains; Boca Raton: CRC Press; 1997 pp. 367–381.

[77] Webb GJW, Manolis SC. Crocodylus johnstoni in the McKinlay River Area N. T, V.* abnormalities and injuries. Aust Wildlife Res. 1983;10(2): 407–420.

[78] Avilla LS, Fernandes R, Ramos DFB. Bite marks on a crocodylomorph from the Upper Cretaceous of Brazil: evidence of social behavior? J Vertebr Paleontol. 2004;24(4): 971–973.

[79] Katsura Y. Paleopathology of Toyotamaphimeis machikanensis (Diapsida, Crocodylia) from the Middle Pleistocene of central Japan. Hist Biol. 2004;16(2): 93–97.

[80] Sereno P, Novas F. The skull and neck of the basal theropod Herrerasaurus ischigualastensis. J Vertebr Paleontol. 1993;13: 451–476.

[81] Tanke DH, Currie PJ. Head-biting behavior in theropod dinosaurs: paleopathological evidence. Gaia. 1998;15: 167–184.

[82] Rootes WL, Chabreck RH. 1993 Cannibalism in the American alligator. Herpetologica. 1993: 99–107.

[83] Amstrup SC, Stirling I, Smith TS, Perham C, Thiemann GW. Recent observations of intraspecific predation and cannibalism among polar bears in the southern Beaufort Sea. Polar Biol. 2006;29(11): 997.

[84] Galentine SP, Swift PK. Intraspecific killing among mountain lions (Puma concolor). Southwestern Nat. 2007;52(1): 161–165.

[85] Hone DW, Tanke DH. Pre-and postmortem tyrannosaurid bite marks on the remains of Daspletosaurus (Tyrannosaurinae: Theropoda) from Dinosaur Provincial Park, Alberta, Canada. PeerJ. 2015 4 9;3:e885 10.7717/peerj.885 25870775 PMC4393819

[86] Roach BT, Brinkman DL. A reevaluation of cooperative pack hunting and gregariousness in *Deinonychus antirrhopus* and other nonavian theropod dinosaurs. Bulletin of the Peabody Museum of Natural History. 2007 4;48(1):103–38.

