# Correction: High frequencies of theropod bite marks provide evidence for feeding, scavenging, and possible cannibalism in a stressed Late Jurassic ecosystem

**DOI:** 10.1371/journal.pone.0315198

**Published:** 2024-12-04

**Authors:** Stephanie K. Drumheller, Julia B. McHugh, Miriam Kane, Anja Riedel, Domenic C. D’Amore

In Table 1, the six linear equations are incorrect. Please see the correct Table 1 here.

**Table 1:** Linear equations used on denticle spacing. The symbol “y” represents the average denticle width of a given theropod tooth for either carina, and “x” represents the natural-logarithm adjusted body size measurement. Striation widths were plugged in as “y” for tooth marked fossils.

Carina	Equation	Measurement
mesial	y = 0.1586x-0.0400	Tooth crown base length (mm)
mesial	y = 0.1725x+0.4588	Skull length (m)
mesial	y = 0.2007x+0.0155	Body length (m)
distal	y = 0.1259x+0.0523	Tooth crown base length (mm)
distal	y = 0.1397x+0.4332	Skull length (m)
distal	y = 0.1642x+0.0689	Body length (m)
